# Interpretable Machine Learning for Predicting Suboptimal 12-Month Growth Response to Recombinant Human Growth Hormone in Children with Idiopathic Short Stature: A Dual-Center External Validation Study

**DOI:** 10.3390/diagnostics16142227

**Published:** 2026-07-16

**Authors:** Chuanyu Yang, Yifeng Shao, Chengyang Jiang, Runmin Zhang, Jian Wang, Xinlin Chen, Yuyuan Zeng, Qi An, Nan Peng, Xi Wang, Bo Zhou, Jianhong Wang, Lin Wang

**Affiliations:** 1 Capital Institute of Pediatrics, Chinese Academy of Medical Sciences & Peking Union Medical College, Beijing 100020, China; yangchuanyu1234@163.com (C.Y.); b2023032011@student.pumc.edu.cn (Y.S.); jiangchengyang1@163.com (C.J.); xinlin_chen@student.pumc.edu.cn (X.C.); b2024032010@student.pumc.edu.cn (Y.Z.); pengnan_727@163.com (N.P.); 2 Department of Child Health Care, Capital Center for Children’s Health, Capital Medical University, Beijing 100020, China; anqi20190220@163.com (Q.A.); 15910683272@163.com (X.W.); bobo-0207@hotmail.com (B.Z.); wja03073@btch.edu.cn (J.W.); 3 Department of General Surgery, Capital Center for Children’s Health, Capital Medical University, Beijing 100020, China; 4 China United Network Communications Group, Beijing Branch, Beijing 100009, China; zhangrm50@chinaunicom.cn; 5 Department of Pediatrics, Beijing Tsinghua Changgung Hospital, School of Clinical Medicine, Tsinghua Medicine, Tsinghua University, Beijing 102218, China; child811117@163.com

**Keywords:** idiopathic short stature, machine learning, clinical prediction model, recombinant human growth hormone, interpretability

## Abstract

**Background/Objectives**: Individual responses to recombinant human growth hormone (rhGH) therapy in children with idiopathic short stature (ISS) vary substantially, limiting pretreatment decision-making. This study aimed to develop and externally validate an interpretable machine learning model for predicting suboptimal 12-month growth response to rhGH therapy. **Methods**: In this retrospective dual-center study, 901 children from Center 1 were used for model development and internal testing, and 51 children from Center 2 formed an independent external validation cohort. Routinely collected baseline demographic, laboratory, hormonal, radiographic, and family-history variables were used to develop multiple machine learning models. A soft-voting ensemble classifier was constructed and interpreted using SHapley Additive exPlanations (SHAP). The primary outcome was suboptimal growth response, defined as failure to achieve a height gain of at least 0.5 standard deviation score after 12 months of treatment. **Results**: The optimized ensemble model showed strong discrimination in the internal test set, with an area under the receiver operating characteristic curve of 0.927, and maintained robust performance in the external validation cohort, with an AUC of 0.897. SHAP analysis identified luteinizing hormone, body mass index, TW3 RUS bone age, and insulin-like growth factor 1 as the leading contributors to predicted suboptimal-response risk. **Conclusions**: An interpretable ensemble machine learning model based on routinely available pretreatment data can predict suboptimal short-term rhGH response in children with ISS and may support individualized risk stratification in pediatric endocrine practice. Clinical trial registration was not required because this was a retrospective analysis.

## 1. Introduction

Idiopathic short stature (ISS) is a prevalent growth disorder in pediatrics, defined as a height > 2 SDS below the age- and sex-matched mean (or below the third percentile) in the absence of systemic, endocrine, chromosomal, or metabolic pathology [1,2]. Epidemiological data indicate that ISS accounts for 60–80% of children presenting with short stature, making it a primary focus of growth evaluation in pediatric endocrinology [2,3].

Recombinant human growth hormone (rhGH) is widely recommended as first-line therapy for ISS, but clinical responses are markedly heterogeneous. Traditional predictors—including baseline height SDS, bone age delay, IGF-1 levels, and parental target height—only explain part of this variability [4,5]. The complex interplay of these factors hinders clinicians’ ability to anticipate individual treatment efficacy before initiating therapy. Notably, registry data have shown that both age at treatment initiation and first-year height response (Δ height SDS) strongly influence near-adult height [6,7]. Given the high cost of rhGH therapy and potential burden of side effects [8,9], precise prediction of individual response is clinically critical.

Traditional multivariable regression models for rhGH response prediction often underperform, as they rely on a limited set of preselected clinical variables and fail to capture complex, nonlinear interdependencies between anthropometric, metabolic, and imaging data [10]. In contrast, machine learning (ML) approaches can integrate high-dimensional data and deliver substantially greater predictive accuracy [11,12]. ML has already been applied in pediatric endocrinology for diagnostic support, such as distinguishing growth hormone deficiency (GHD) from ISS [13,14]. However, few studies have focused on predicting rhGH treatment efficacy in ISS, and externally validated interpretable ML models remain limited.

To address this gap, we developed an interpretable, non-invasive ML model based on baseline clinical and laboratory data to predict suboptimal 12-month growth response in children with ISS receiving rhGH. We applied SHapley Additive exPlanations (SHAP) to quantify the contribution of each predictor to predicted suboptimal-response risk, thereby offering transparent insights into factors associated with inadequate growth, and externally validated the model using patients from a second center to support clinical judgment in pediatric endocrinology.

## 2. Materials and Methods

### 2.1. Study Design and Participants

**Figure 1:**
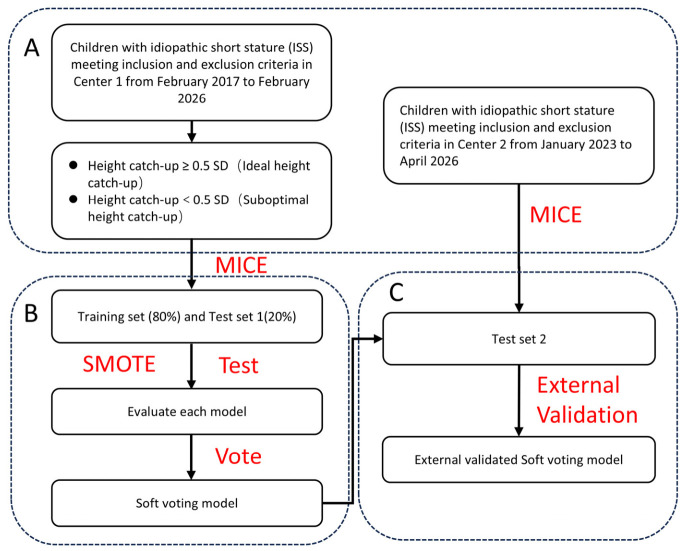
Flow diagram showing the study protocol implementation. (**A**) Patient enrollment, data collection, and preprocessing; (**B**) model development and internal validation in Center 1; (**C**) external validation in Center 2.

This study analyzed data of children with ISS who received rhGH treatment. Eligible patients were retrospectively identified from the Department of Child Health Care of Capital Center for Children’s Health, Capital Medical University (Center 1), with follow-up data available through February 2026, and from the Department of Pediatrics of Beijing Tsinghua Changgung Hospital (Center 2), with follow-up data available through April 2026. Only children who had completed at least 12 months of continuous rhGH therapy and had available 12-month outcome data were included. Baseline demographic, laboratory, and imaging data were extracted from the Hospital Information System (HIS) and Laboratory Information Management System (LIS). The study protocol is shown in Figure 1.

Inclusion criteria were: (1) height below the 3rd percentile for age and sex at treatment onset (Chinese Pediatric Growth Standard); (2) available 12-month growth outcome after continuous rhGH therapy; and (3) absence of comorbidities likely to affect growth (e.g., GH deficiency, overt thyroid dysfunction, severe cardiac, hepatic, or renal disease, chromosomal abnormalities, or skeletal disorders).

Exclusion criteria were: (1) >30% of key data missing; and (2) poor treatment adherence (defined as <90% of prescribed injections or irregular follow-up visits, reported by parents).

All children received subcutaneous rhGH according to routine clinical practice and product instructions. Short-acting rhGH was initially prescribed at 0.15 IU/kg/day, whereas long-acting rhGH was initially prescribed at 0.2 mg/kg/week. Follow-up visits were scheduled every 3 months, and doses were adjusted when appropriate according to body weight, growth response, IGF-1 levels, and safety monitoring. The dosing strategy and follow-up principles were consistent between the two centers. The choice of short-acting rhGH, long-acting rhGH, or mixed use during follow-up was mainly determined by family preference and cost considerations rather than by center-specific physician assignment. Treatment adherence was assessed from follow-up records and caregiver reports, and children with poor adherence (<90% of prescribed injections or irregular follow-up visits) were excluded.

This study was approved by the Ethics Committee of Capital Center for Children’s Health, Capital Medical University (Approval No.: DWLL2026004) and the Ethics Committee of Beijing Tsinghua Changgung Hospital (Approval No.: 26094-6-01). Patient consent was waived by the ethics committees.

### 2.2. Data Collection and Preprocessing

Comprehensive baseline characteristics were collected, including: (1) demographics; (2) hematology; (3) biochemistry; (4) metabolic/glucose indices; (5) electrolytes and iron status; (6) hormonal markers; (7) imaging; (8) perinatal/family history; and (9) other biomarkers.

Categorical non-ordinal variables (e.g., sex, preterm birth) were transformed using one-hot encoding. Continuous variables were standardized (z-score normalization) to ensure consistent scaling across ML models.

### 2.3. Outcome Definition

Suboptimal growth response was defined a priori as a height SDS gain < 0.5 after 12 months of continuous rhGH therapy. This cutoff was selected because first-year height SDS gain is commonly used to evaluate rhGH treatment response, although no single universally accepted ISS-specific threshold exists across all guidelines. Height SDS was calculated using the Chinese Pediatric Growth Standard [1,2]. Height and weight were measured with a calibrated pediatric anthropometer (Shanghai Betterren Medical Technology Co., Ltd., Shanghai, China, model: customized BG-A30) with a precision of ±0.1 cm (height) and ±0.05 kg (weight). Measurements were performed by trained nurses, with 2 repeated measurements per visit (mean value used for analysis).

### 2.4. Handling of Missing Data

Variables with more than 30% missingness were excluded. For the remaining features, missing values were imputed using multivariate multiple imputation via chained equations (MICE).

### 2.5. Model Development and Statistical Analysis

Normality was assessed using the D’Agostino–Pearson test. Normally distributed continuous variables were presented as mean ± SD, and non-normally distributed variables were presented as median (interquartile range). Between-group comparisons were conducted using independent t-tests for parametric data, Mann–Whitney U tests for nonparametric data, and Pearson’s chi-squared or Fisher’s exact test for categorical variables, depending on expected frequencies.

Our data were partitioned into a training set (80%) and a held-out test set (20%), the latter remaining blinded throughout model development. Feature selection proceeded through a multi-stage strategy: first, we computed Spearman’s rank correlation on the training set to remove highly correlated variables (ρ > 0.8) to mitigate multicollinearity; second, we employed recursive feature elimination based on a random forest regressor for variable ranking. Clinical experts oversaw the process to ensure that features with known pathophysiological or clinical relevance were retained, even if eliminated by automated procedures.

Using the selected features, we developed multiple predictive models: logistic regression, support vector machine, random forest, gradient boosting machines (including XGBoost and LightGBM), AdaBoost, and K-nearest neighbors. These algorithms were selected to represent both linear and nonlinear modeling strategies, enabling comparison across different levels of model complexity. Hyperparameters and ensemble weights were optimized within the training data using stratified 5-fold cross-validation and grid search. The final model was selected based on internal validation performance, and the trained model was subsequently evaluated in the held-out internal test set and independent external validation cohort. A soft-voting ensemble classifier aggregated probabilistic outputs from the selected base models. To address class imbalance, we applied the Synthetic Minority Over-sampling Technique (SMOTE) to training folds only, ensuring no data leakage into the test set. Statistical and machine-learning analyses were implemented in Python (Python Software Foundation, Wilmington, DE, USA) using open-source packages, including scikit-learn, XGBoost, LightGBM, SHAP, pandas, NumPy, SciPy, and imbalanced-learn.

Model performance was assessed using six metrics: accuracy, sensitivity (recall), specificity, positive predictive value, negative predictive value, and F1 score (the harmonic mean of precision and recall). Sensitivity was prioritized as the primary metric because failing to identify children with a suboptimal response may lead to unnecessary treatment burden and delayed alternative management. Calibration was visually assessed using calibration plots, and overall prediction error was quantified using the Brier score. Confidence intervals (95%) for each metric were estimated by nonparametric bootstrap (2000 resamples).

### 2.6. Model Interpretability

SHapley Additive exPlanations (SHAP) were used to interpret the ensemble model. Feature importance was ranked by mean absolute SHAP value, and the top contributing features were further analyzed. The direction and magnitude of each feature’s effect were interpreted in relation to the predicted probability of suboptimal 12-month growth response.

### 2.7. External Validation

To assess the generalizability of the model, we validated it using an independent dataset collected from a second medical center (Center 2, *n* = 51). This dataset was processed using the same preprocessing pipeline (imputation and scaling parameters derived from Center 1) to ensure consistency. The trained ensemble model was applied directly to Center 2 data without retraining.

This study was reported with reference to the STROBE and TRIPOD-AI reporting recommendations for observational studies and prediction model research.

## 3. Results

### 3.1. Feature Selection Results

According to the inclusion and exclusion criteria, a total of 901 subjects from Center 1 and 51 subjects from Center 2 were enrolled for subsequent analysis.

The Center 1 cohort was randomly split into a training set (*n* = 720, including 174 children with suboptimal response) and an internal test set (*n* = 181, including 44 children with suboptimal response). The external validation cohort included 51 children from Center 2, of whom 14 had a suboptimal response.

At baseline, after the multi-stage feature selection strategy, 49 key predictors were retained. Of 901 children, 218 (24.2%) did not achieve a height gain of ≥0.5 SDS after 12 months of continuous rhGH therapy (Table 1). Feature categories are provided in Supplementary Table S1 to clarify the clinical domains represented by the selected predictors, and the correlation structure among selected features is shown in Figure S1.

### 3.2. Model Performance

**Figure 2:**
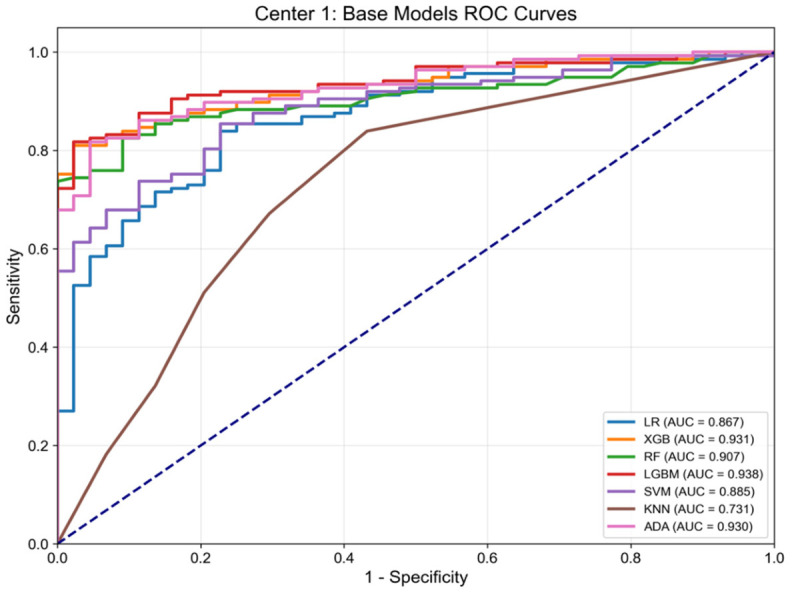
Receiver operating characteristic (ROC) curves of seven base machine learning models.

**Figure 3:**
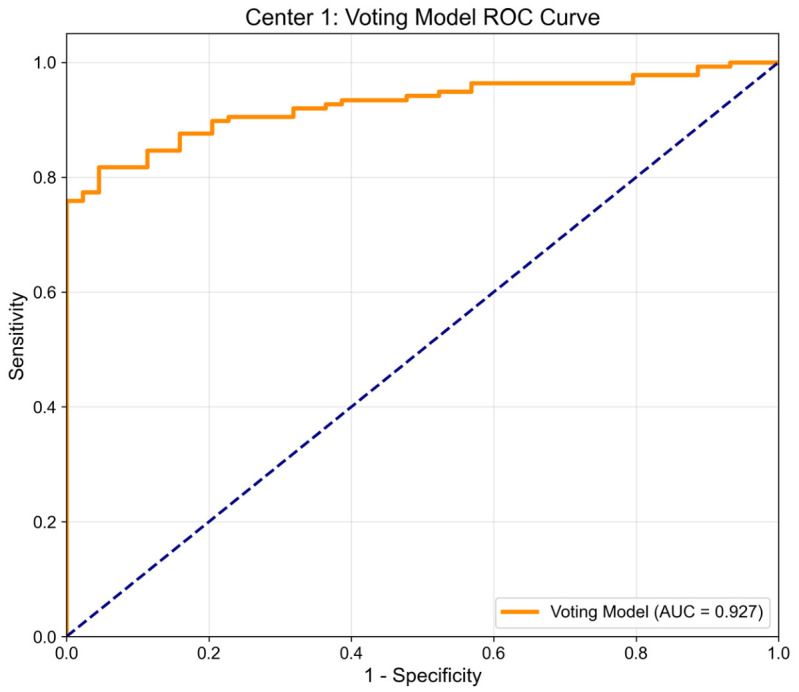
Receiver operating characteristic (ROC) curve of the soft-voting ensemble classifier.

The ROC curves of the seven base machine-learning models are shown in Figure 2. In the internal test set, the logistic regression (LR) model achieved an AUC of 0.867. Among nonlinear models, LightGBM achieved the highest AUC (0.938). The optimized soft-voting ensemble classifier provided a balanced and robust performance profile, with AUC = 0.927 (Figure 3), recall = 0.871, specificity = 0.829, and F1 score = 0.907. The confusion matrix of the optimized ensemble classifier is provided in Figure S2.

### 3.3. Interpretability (SHAP) Analysis

SHAP analysis of the ensemble model identified the top four contributors to predicted suboptimal-response risk: LH (0.16), BMI (0.06), TW3 RUS bone age (0.04), and IGF-1 (0.03) (Table S1).

**Figure 4:**
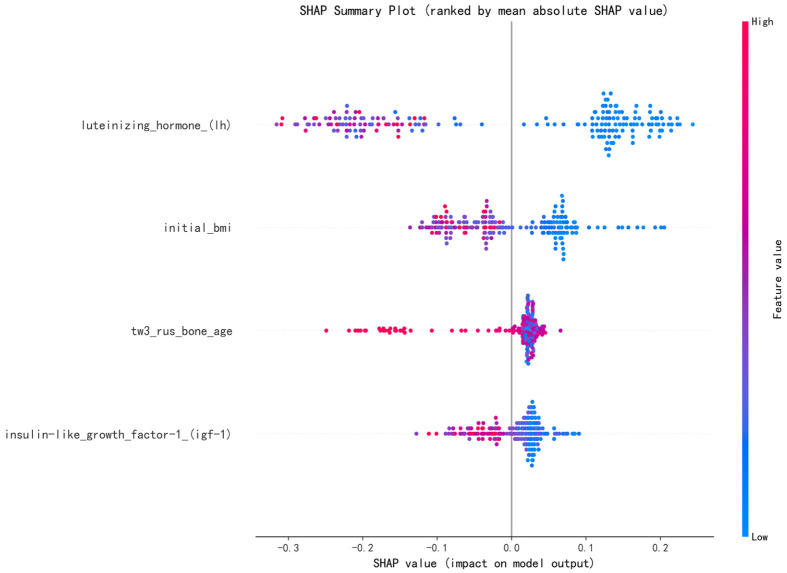
SHAP-based interpretation of the soft-voting ensemble classifier: the top four features contributing to predicted suboptimal 12-month growth response.

**Figure 5:**
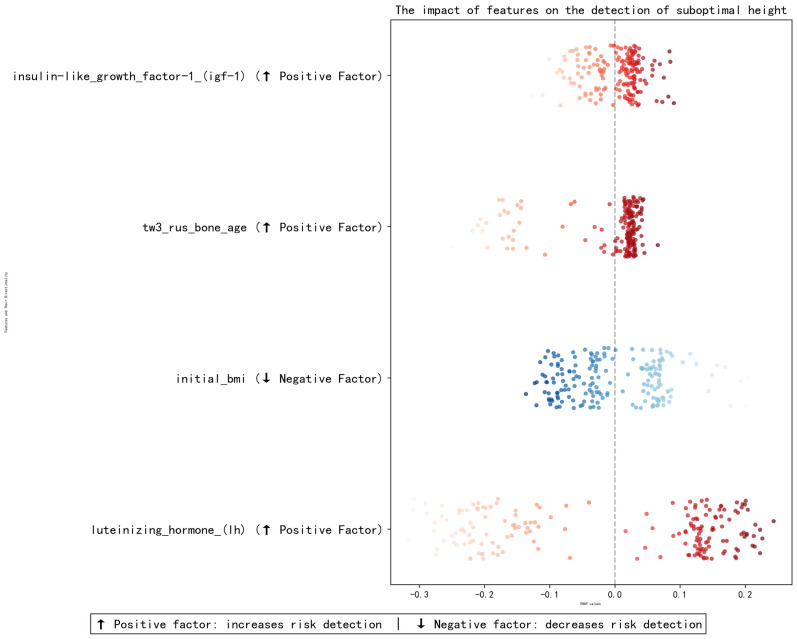
Directionality of each feature’s impact on the predicted suboptimal 12-month growth response. Horizontal position indicates the direction and magnitude of the feature effect on predicted suboptimal-response risk; color represents the relative feature value, with red indicating higher values and blue indicating lower values.

**Figure 6:**
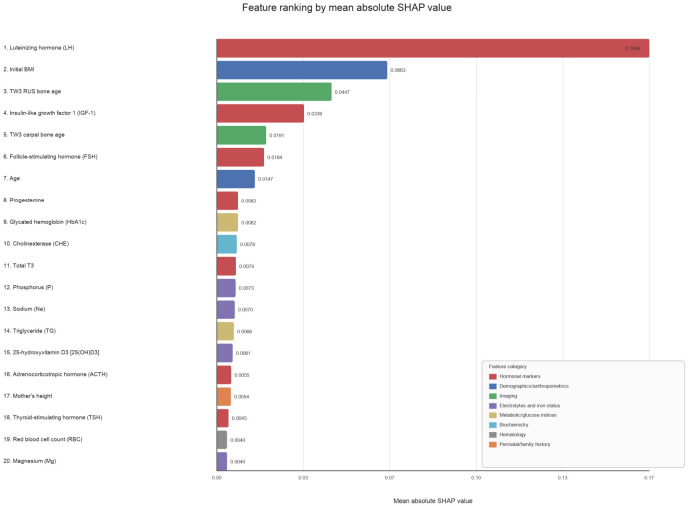
Feature ranking by mean absolute SHAP value. Bars are colored by feature category.

SHAP summary plots further confirmed that these four predictors contributed most to interindividual variability in predicted suboptimal-response risk (Figure 4). Partial dependence plots showed that higher LH, TW3 RUS bone age, and IGF-1 were associated with a higher predicted probability of suboptimal 12-month response. In contrast, higher BMI was associated with a lower predicted probability of suboptimal response (Figure 5). The ranked contribution of the top predictors based on mean absolute SHAP values is shown in Figure 6.

### 3.4. External Validation Performance

**Figure 7:**
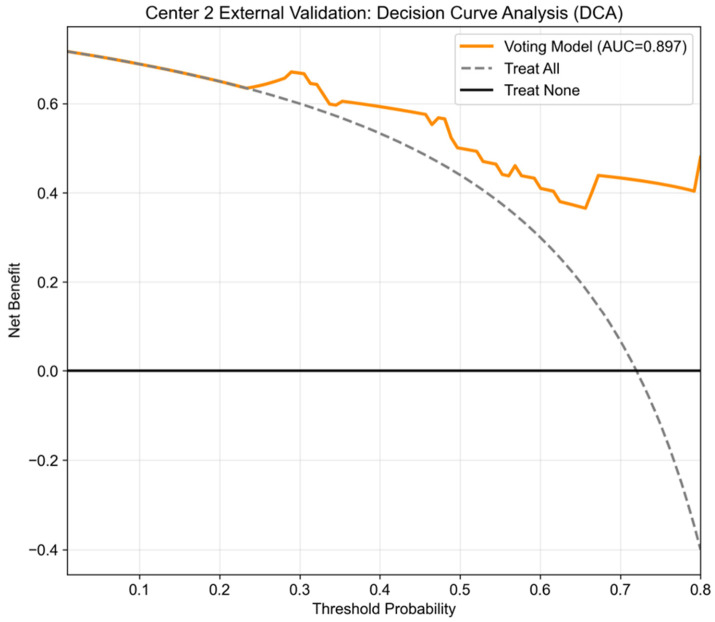
Performance of the soft-voting ensemble classifier in external validation and decision curve analysis (DCA).

Preliminary external validation suggested acceptable transportability to a second clinical setting. The model achieved an AUC of 0.897 in the external validation cohort. Calibration analysis showed reasonable agreement between predicted probabilities and observed outcomes (Brier score = 0.132), and decision curve analysis suggested potential net clinical benefit across a range of threshold probabilities (Figure 7).

## 4. Discussion

### 4.1. Principal Findings

This study developed and validated an interpretable machine learning model that integrates routine clinical, hormonal, and radiographic indicators to predict suboptimal 12-month growth response to recombinant human growth hormone (rhGH) therapy in children with idiopathic short stature. The model was specifically designed to identify children at risk of an inadequate short-term response before treatment initiation. The ensemble model demonstrated strong discrimination (AUC: 0.927; recall: 0.871), and SHAP analysis consistently identified LH, BMI, TW3-RUS bone age, and IGF-1 as the most influential predictors of suboptimal-response risk.

Higher LH levels and more advanced TW3 RUS bone age may reflect earlier pubertal activation or accelerated skeletal maturation, both of which can reduce remaining growth potential and thereby increase the risk of a suboptimal 12-month response. Higher baseline IGF-1 may indicate more advanced maturation, altered GH-IGF axis sensitivity, or a smaller capacity for further rhGH-induced IGF-1 response, rather than directly proving IGF-1 resistance. The association between higher BMI and lower predicted suboptimal-response risk may reflect better nutritional reserve, although BMI may also act as a proxy for pubertal or metabolic status. These interpretations should be viewed as biologically plausible explanations of model behavior rather than evidence of causal mechanisms. SHAP values quantify feature contributions to model predictions and do not establish causal relationships.

Collectively, these findings support the feasibility of using pretreatment parameters to identify children at elevated risk of a suboptimal 12-month response. The model’s interpretability further enhances its clinical relevance by clarifying the direction and relative magnitude of predictor contributions to predicted suboptimal-response risk.

### 4.2. Comparison with Prior Work

The findings align with previous large-scale cohort analyses showing that growth response is shaped by baseline anthropometric and endocrine characteristics. Prior studies highlighted age, distance from target height, and baseline height SDS as important determinants of first-year response [4,15]. The positive association between higher BMI and improved response mirrors observations in the LG Growth Study analyses, particularly among children with idiopathic short stature or partial GH deficiency [16,17]. The inverse association between baseline IGF-1 and growth response is consistent with pharmacodynamic studies indicating that higher IGF-1 may reflect reduced anabolic reserve or diminished GH responsiveness [18]. In addition, the adoption of SHAP to enhance transparency corresponds with an expanding body of ML-based endocrine research that prioritizes explainability to improve clinical acceptance [11,19]. Together, this study advances earlier work by integrating radiographic, hormonal, and clinical domains into a unified, interpretable prediction framework.

### 4.3. Clinical Implications

The model provides several potential implications for clinical practice. It should not be used as a stand-alone tool to withhold or delay rhGH treatment. Instead, the predicted risk can support shared decision-making by identifying children who may benefit from more intensive counseling, adherence support, nutritional assessment, pubertal evaluation, and early response monitoring. Any dose adjustment should remain based on clinical assessment, growth response, IGF-1 monitoring, and safety evaluation. In decision curve analysis, the threshold probability represents the predicted risk level at which clinicians and families would consider additional management actions worthwhile. In this setting, lower thresholds may favor sensitivity and early counseling, whereas higher thresholds may identify children who warrant more intensive follow-up, adherence support, nutritional/pubertal reassessment, and early evaluation of treatment response. These thresholds should not be used to deny rhGH therapy and require prospective validation before implementation.

### 4.4. Limitations and Future Directions

Our study has several limitations. First, although the model was developed at a single center, this limitation was partially mitigated by validation in an external cohort. However, the external validation cohort was relatively small, and the transportability of the model should be further assessed in larger multicenter cohorts. Second, because the model was developed among children who completed at least 12 months of rhGH therapy and had available follow-up data, children who discontinued treatment early or had poor adherence were not represented. This selection may underestimate the burden of poor response in real-world practice and limits applicability to non-adherent or early-discontinuation populations. Third, genomic, nutritional, and lifestyle data were unavailable, and these variables may further improve prediction. Fourth, Tanner stage and pubertal progression during follow-up were not consistently available in the retrospective records and could not be incorporated into the model. Although LH, FSH, and TW3 bone age provide indirect information on pubertal maturation, they cannot fully replace standardized pubertal staging. Fifth, adherence was based on caregiver self-report, which may be subject to bias. Future prospective studies should incorporate adherence trajectories, early discontinuation, standardized pubertal staging, and electronic injection-device records when available.

Future research should prioritize several key directions: first, external validation of the model in multicenter, multiethnic cohorts to rigorously assess its generalizability across diverse patient groups; second, integration of the model into Hospital Information System (HIS)- and Laboratory Information Management System (LIS)-embedded clinical decision support systems to enable real-time, point-of-care application; and finally, incorporation of multi-omics data and longer-term growth outcomes, including two-year response and near-adult height, into future models [20], followed by well-designed prospective clinical trials to determine whether the model improves clinical decision-making in practice.

## 5. Conclusions

This study developed an interpretable machine learning model for predicting suboptimal 12-month growth response to rhGH therapy in children with ISS. The model showed strong discrimination in the internal test set (AUC = 0.927) and acceptable transportability in an independent external validation cohort (AUC = 0.897). Feature-importance analysis indicated that hormonal markers, imaging-derived skeletal maturity measures, and demographic/anthropometric factors were the most informative domains, with LH, BMI, TW3 RUS bone age, and IGF-1 contributing most strongly to predicted suboptimal-response risk. These findings suggest that pretreatment endocrine status, skeletal maturation, and nutritional/anthropometric status jointly inform individualized risk stratification before rhGH treatment. Future multicenter prospective studies should validate the model, refine clinically actionable risk thresholds, and evaluate whether model-guided monitoring improves long-term growth outcomes.

## Figures and Tables

**Table 1:** Baseline characteristics and selected features used for model construction.

Characteristic	Did Not Achieve ≥0.5 SDS Gain (*n* = 218)	Achieved ≥0.5 SDS Gain (*n* = 683)	*p*
Sex (encoded as 0/1)	1.00 (0.00, 1.00)	0.00 (0.00, 1.00)	<0.001
Age (years)	10.84 (9.00, 12.51)	8.65 (6.31, 10.30)	<0.001
Initial BMI (kg/m^2^)	18.57 (17.00, 20.62)	15.79 (14.54, 17.59)	<0.001
Mean corpuscular hemoglobin concentration (MCHC)	338.67 ± 11.07	338.12 ± 10.99	0.523
Mean corpuscular hemoglobin (MCH)	28.45 (27.80, 29.38)	28.00 (27.20, 28.80)	<0.001
Mean corpuscular volume (MCV)	84.23 ± 3.88	82.80 (80.20, 85.30)	<0.001
Red blood cell count (RBC)	4.73 (4.53, 4.98)	4.69 (4.46, 4.88)	<0.05
Hematocrit (HCT)	0.40 (0.38, 0.42)	0.39 ± 0.03	<0.001
Hemoglobin (Hb)	135.00 (129.00, 142.00)	131.01 ± 8.62	<0.001
Total iron-binding capacity (TIBC)	63.80 (63.52, 67.20)	63.80 (60.80, 66.60)	0.069
Cholinesterase (CHE)	9.36 (9.02, 9.66)	9.36 (8.73, 10.05)	0.665
Serum iron (Fe)	16.65 (15.20, 19.45)	16.65 (14.30, 19.10)	0.285
Magnesium (Mg)	0.86 ± 0.05	0.86 ± 0.05	0.307
Thyroid-stimulating hormone (TSH)	1.75 (1.31, 2.33)	1.89 (1.42, 2.54)	<0.05
Total T3	2.01 (1.81, 2.17)	2.03 (1.88, 2.22)	<0.01
Total T4	99.50 (89.22, 110.47)	104.30 (93.20, 115.70)	<0.001
Total cholesterol (TC)	4.17 (3.67, 4.67)	4.28 (3.83, 4.71)	0.079
Total protein (TP)	72.56 ± 4.30	71.85 ± 4.19	<0.05
Free T3	5.62 ± 0.61	5.68 (5.29, 6.07)	0.105
Free T4	13.35 ± 1.43	13.70 (12.80, 14.70)	<0.01
Triglyceride (TG)	0.72 (0.65, 0.98)	0.72 (0.60, 0.90)	<0.05
Alkaline phosphatase (ALP)	252.00 (215.25, 319.50)	253.00 (209.00, 302.00)	0.132
Phosphorus (P)	1.71 ± 0.17	1.71 (1.60, 1.82)	0.701
Glucose (GLU)	4.86 ± 0.36	4.78 (4.52, 5.00)	<0.001
Calcium (Ca)	2.46 (2.39, 2.54)	2.47 ± 0.09	0.662
Sodium (Na)	0.53 (0.28, 0.75)	0.48 (0.28, 0.73)	0.141
Potassium (K)	0.50 (0.24, 0.73)	0.49 (0.23, 0.75)	0.659
Unsaturated iron-binding capacity (UIBC)	46.80 (44.82, 49.35)	46.80 (42.80, 50.50)	0.961
Globulin	25.95 ± 3.26	25.20 (23.10, 27.50)	<0.05
Albumin-globulin ratio (A/G ratio)	1.80 (1.70, 2.00)	1.80 (1.70, 2.00)	<0.05
Albumin (ALB)	46.60 (45.30, 48.58)	46.60 (45.00, 48.20)	0.384
25-hydroxyvitamin D3 [25(OH)D3]	50.57 (48.59, 52.43)	50.57 (50.57, 51.63)	0.718
Insulin-like growth factor 1 (IGF-1)	290.00 (246.00, 383.75)	179.00 (116.00, 265.00)	<0.001
Post-exercise growth hormone (post-exercise GH)	7.22 (7.22, 7.22)	7.22 (7.22, 7.22)	0.922
Glycated hemoglobin (HbA1c)	5.40 (5.20, 5.50)	5.30 (5.20, 5.50)	<0.05
Adrenocorticotropic hormone (ACTH)	19.50 (17.60, 25.15)	19.50 (15.30, 25.10)	0.301
Growth hormone (GH)	1.16 (1.16, 1.16)	1.16 (1.10, 1.21)	0.577
Cortisol (COR)	7.51 (6.25, 8.29)	7.51 (6.55, 8.99)	0.223
Follicle-stimulating hormone (FSH)	3.45 (2.28, 5.11)	2.00 (1.27, 3.28)	<0.001
Luteinizing hormone (LH)	2.31 (1.29, 3.17)	0.57 (0.13, 1.11)	<0.001
Prolactin (PRL)	8.77 (6.62, 12.44)	8.33 (6.33, 11.76)	0.157
Insulin	9.25 (7.90, 13.80)	9.25 (6.80, 10.35)	<0.001
Progesterone	0.58 (0.43, 0.95)	0.58 (0.33, 0.84)	<0.05
C-peptide	1.74 (1.74, 2.12)	1.74 (1.54, 1.88)	<0.01
TW3 RUS bone age	149.50 (122.00, 164.00)	116.00 (78.00, 132.00)	<0.001
TW3 carpal bone age	136.00 (114.00, 153.00)	110.00 (78.00, 127.00)	<0.001
Father’s height (cm)	170.00 (170.00, 170.00)	170.00 (168.00, 172.00)	0.717
Mother’s height (cm)	158.00 (158.00, 160.00)	158.00 (156.00, 160.00)	0.836
Premature birth (encoded as 0/1)	0.00 (0.00, 0.00)	0.00 (0.00, 0.00)	0.253

Note: Sex and premature birth were encoded as 0/1 in the modeling dataset. Continuous variables are presented as mean ± SD or median (interquartile range), as appropriate. *n* denotes the number of participants, and *p* denotes the *p*-value.

## Data Availability

The de-identified data generated and/or analyzed in this study are not publicly available due to privacy and ethical restrictions, but are available from the corresponding author on reasonable request and with appropriate ethical approval. The analysis code and trained model can be made available from the corresponding author on reasonable request.

## Supplementary Materials

The following supporting information can be downloaded at: https://www.mdpi.com/article/10.3390/diagnostics16142227/s1. Table S1: Feature-importance ranking with feature categories based on SHAP analysis; Figure S1: Feature heatmap; Figure S2: Confusion matrix.

## Abbreviations

The following abbreviations are used in this manuscript:

ISS	Idiopathic short stature
rhGH	Recombinant human growth hormone
ML	Machine learning
AUC	Area under the curve
SHAP	SHapley Additive exPlanations
LR	Logistic regression
RF	Random forest
XGB	eXtreme gradient boosting
LGBM	Light gradient boosting machine
SVM	Support vector machine
KNN	K-nearest neighbors
SDS	Standard deviation score
IGF-1	Insulin-like growth factor 1
LH	Luteinizing hormone
FSH	Follicle-stimulating hormone
BMI	Body mass index
