# Supplementary figures and images for: Interpretable Machine Learning for Predicting Suboptimal 12-Month Growth Response to Recombinant Human Growth Hormone in Children with Idiopathic Short Stature: A Dual-Center External Validation Study
Source: Diagnostics (Basel). 2026 Jul 16;16(14):2227. doi: 10.3390/diagnostics16142227 (PMC13408863; doi:10.3390/diagnostics16142227)

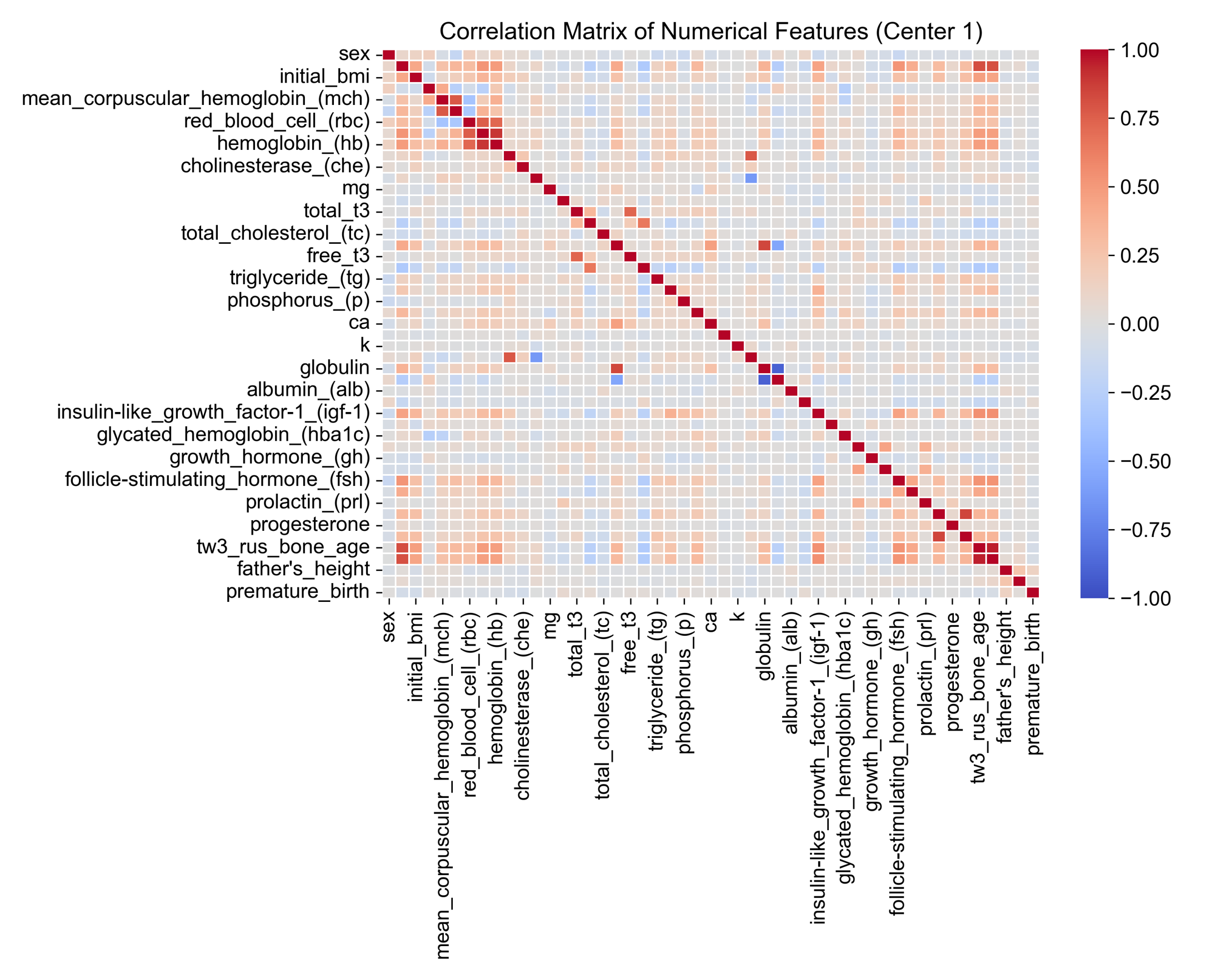

Supplement: Supplementary file 1 [file diagnostics-16-02227-s001.zip › Supplementary materials/S1.tif]

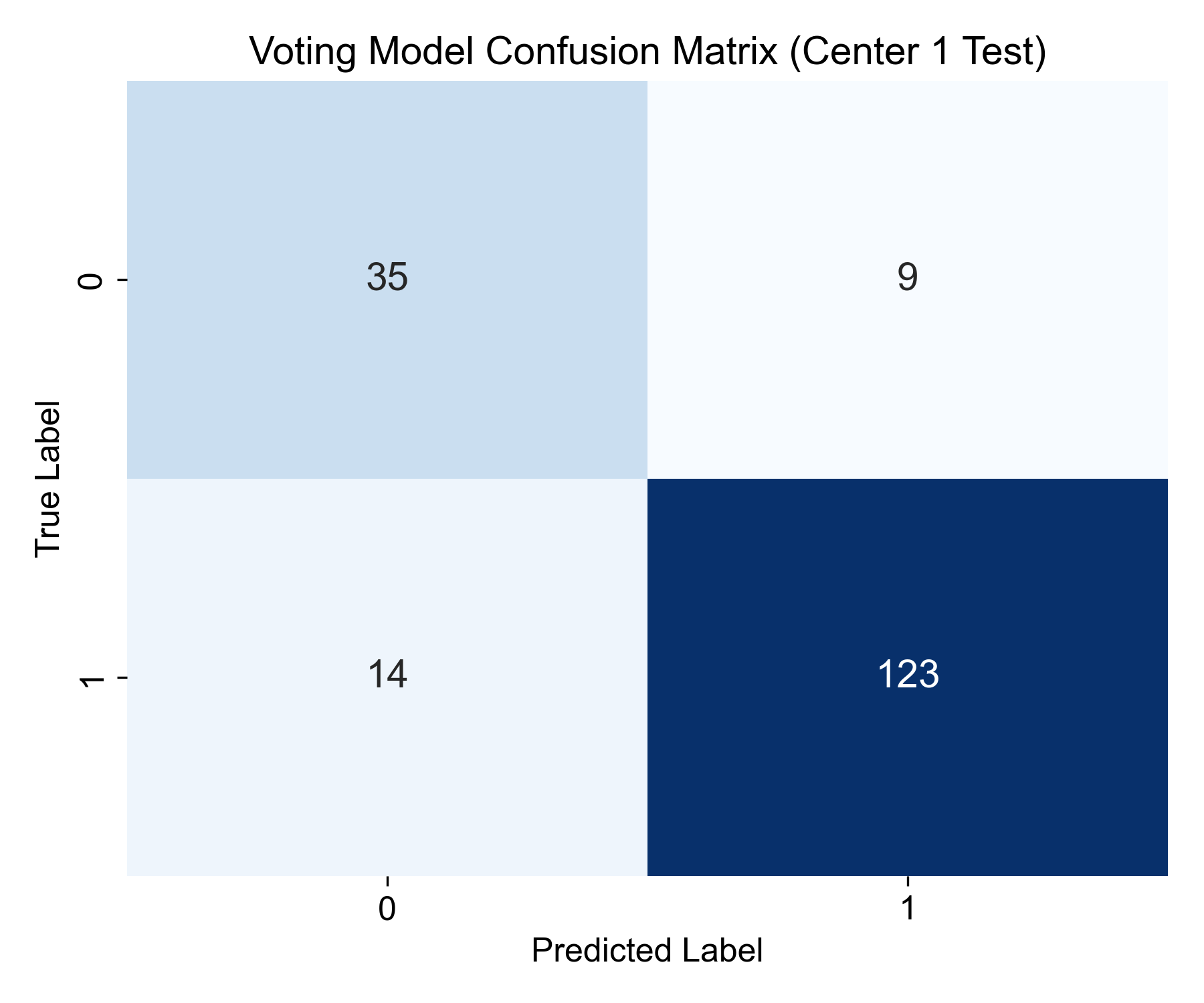

Supplement: Supplementary file 1 [file diagnostics-16-02227-s001.zip › Supplementary materials/S2.tif]
